# Tumor Regression Grade as a Predictor of Adjuvant Therapy Benefits in Esophageal Squamous Cell Carcinoma Patients After Neoadjuvant Therapy

**DOI:** 10.1002/cam4.71166

**Published:** 2025-09-13

**Authors:** Yizhou Huang, Maohui Chen, Yuanpu Wei, Bingqiang Cai, Yongcong Zhang, Chuanquan Lin, Shuliang Zhang, Taidui Zeng, Chun Chen, Bin Zheng

**Affiliations:** ^1^ Department of Thoracic Surgery Fujian Medical University Union Hospital Fuzhou China; ^2^ Key Laboratory of Cardio‐Thoracic Surgery (Fujian Medical University), Fujian Province University Fuzhou China; ^3^ National key Clinical Specialty of Thoracic Surgery Fuzhou China; ^4^ Clinical Research Center for Thoracic Tumors of Fujian Province Fuzhou China; ^5^ Department of Thoracic Surgery The First Affiliated Hospital of Xiamen University Xiamen China; ^6^ Department of Thoracic Surgery Quanzhou First Hospital Fujian China

**Keywords:** adjuvant therapy, esophageal cancer, pathological lymph nodes, survival analysis, tumor regression grade

## Abstract

**Background:**

Neoadjuvant therapy followed by surgery is preferred for locally advanced esophageal squamous cell carcinoma (ESCC), but the necessity of adjuvant therapy remains controversial. Tumor regression grade (TRG) reflects the response to neoadjuvant therapy and may predict patient prognosis, yet its role in guiding adjuvant therapy remains unexplored. This study aimed to explore the role of TRG and other clinical characteristics in predicting the efficacy of postoperative adjuvant therapy in ESCC patients receiving neoadjuvant therapy.

**Methods:**

This study included patients who underwent R0 esophagectomy for thoracic ESCC after neoadjuvant therapy between January 2016 and December 2021 across three high‐volume centers. Patients were assessed by TRG and divided into good responders (TRG 0–1) and poor responders (TRG 2–3).

**Results:**

Among 416 patients with a median follow‐up of 52 months, adjuvant therapy extended median survival by 8 months, which was statistically insignificant (*p* = 0.28). In the TRG 0–1 subgroup, those receiving adjuvant therapy had 3‐year and 5‐year OS rates of 94.6% and 86.8%, compared to 78.8% and 71.6% for the observation group (*p* = 0.02). Multivariable Cox regression showed adjuvant therapy was associated with reduced mortality in the TRG 0–1 (HR 0.32; 95% CI 0.14–0.73; *p* = 0.006), positive lymph nodes (HR 0.53; 95% CI 0.36–0.78; *p* = 0.001), and ypT3‐4 subgroups (HR 0.63; 95% CI 0.43–0.92; *p* = 0.017).

**Conclusions:**

TRG is a promising predictor of the prognostic value of adjuvant therapy in ESCC patients. Patients with a good TRG response, positive lymph nodes, and ypT3‐4 stage benefit from adjuvant therapy.

## 1 Introduction

Esophageal cancer ranks as the eighth most common cancer globally and the sixth leading cause of cancer‐related death [1]. Neoadjuvant therapy (NAT) is the standard treatment for locally advanced esophageal cancer, reducing tumor size, micrometastases, and improving resectability [2, 3]. The National Comprehensive Cancer Network (NCCN) guidelines recommend postoperative observation for patients who achieve complete resection (R0) without prior neoadjuvant chemoradiotherapy, regardless of pathological stage [4]. This recommendation stems from a lack of supporting evidence from randomized controlled trials, leading to inconsistencies in clinical decision‐making. The choice of adjuvant therapy after surgery is typically based on clinical and pathological factors such as tumor stage and lymph node metastasis [5, 6]. Earlier studies have suggested that adjuvant therapy post‐neoadjuvant treatment is particularly beneficial for patients with advanced esophageal cancer who exhibit positive pathological lymph nodes, ypT4 stage, and positive resection margins [7, 8, 9], although these studies often suffer from limitations such as small sample sizes and patient variability.

Tumor regression grade (TRG) is a pathological metric used to evaluate the response of a tumor to NAT by measuring the proportion of remaining viable tumor cells in surgical specimens, thus categorizing patients into different grades [10]. The American Joint Committee on Cancer (AJCC) TRG system ranges from complete pathological response (TRG 0) to poor response (TRG 3) [11]. TRG reflects NAT effectiveness and predicts prognosis [12, 13] and may guide decisions regarding postoperative adjuvant therapy. Research on postoperative treatments for cancers such as gastroesophageal adenocarcinoma and pancreatic ductal adenocarcinoma has demonstrated that positive responses to NAT can inform decisions on adjuvant therapy, potentially leading to better survival outcomes [14, 15]. However, the effectiveness of NAT, particularly the role of TRG in guiding postoperative adjuvant therapy for esophageal squamous cell carcinoma (ESCC), has yet to be definitively confirmed.

This multicenter, retrospective cohort study gathered detailed data on factors that could influence adjuvant therapy and patient outcomes, focusing on the effectiveness of NAT. The study aimed to identify patient subgroups that are likely to benefit from additional adjuvant therapy and those who might not benefit or could even be adversely affected.

## 2 Methods

### 2.1 Study Population and Ethics

**FIGURE 1:**
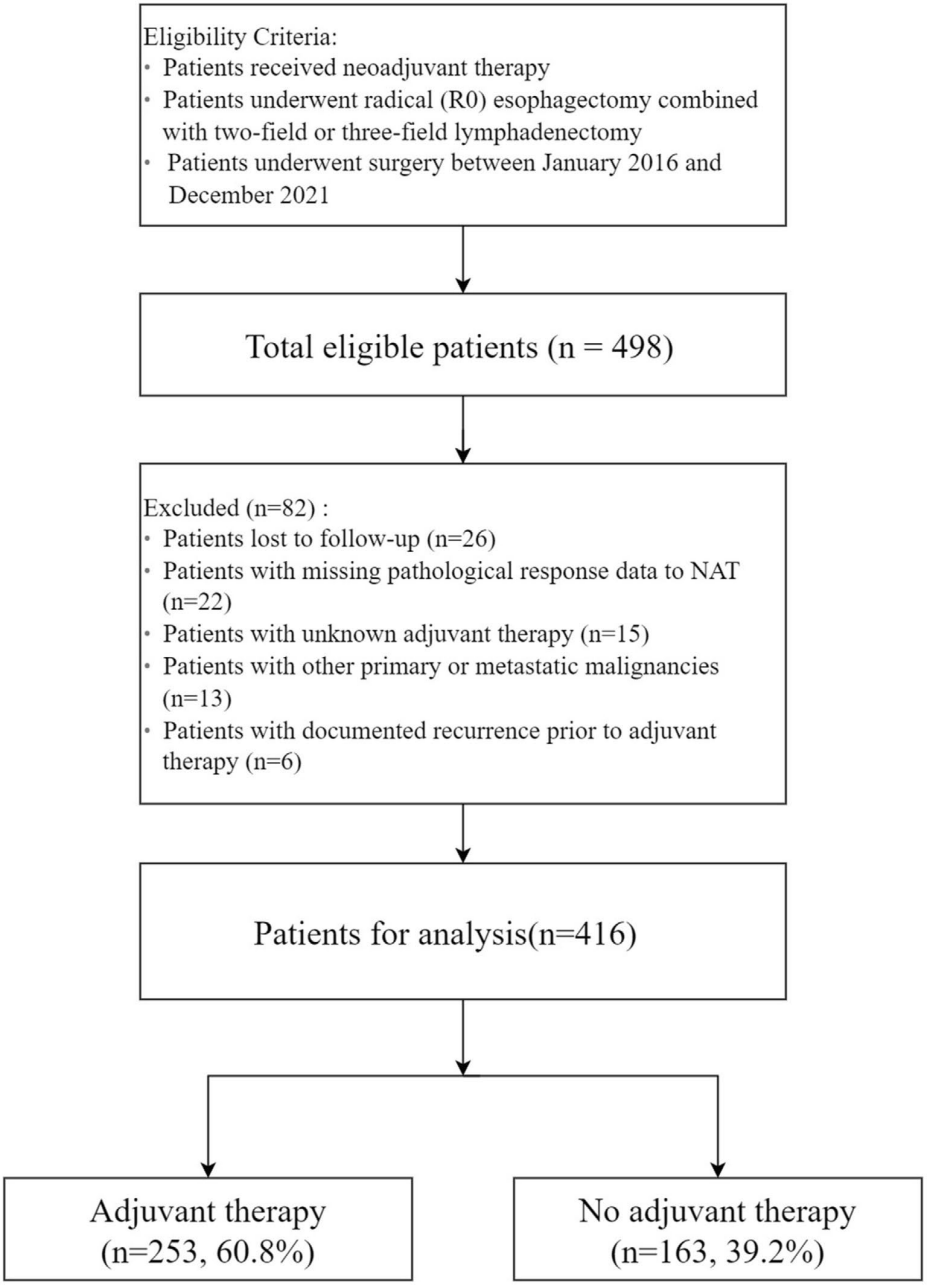
Study flowchart.

This retrospective study collected clinical data and follow‐up results from patients with locally advanced esophageal cancer who underwent radical surgery at three hospitals: Fujian Medical University Union Hospital, the First Affiliated Hospital of Xiamen University, and the Quanzhou First Hospital. The inclusion criteria included patients who: (i) underwent R0 resection with either two‐field or three‐field lymphadenectomy, (ii) received NAT such as preoperative radiotherapy, chemoradiotherapy or combined immunotherapy, and (iii) had ESCC pathology. The exclusion criteria were patients with incomplete clinical data or follow‐up information and those diagnosed with multiple primary malignancies. The cohort creation process is illustrated in Figure 1. This study adhered to the Declaration of Helsinki and received approval from the Ethics Committee of Fujian Medical University Union Hospital (2024KY133).

### 2.2 Variables and Study Outcome

The main variable of interest was adjuvant therapy, and the primary outcome was OS, defined as time from diagnosis until death. We collected data about receipt of adjuvant therapy and classified patients into two groups based on their TRG: good responders (TRG 0–1) and poor responders (TRG 2–3). We collected clinical and demographic data, including age at diagnosis, gender, body mass index (BMI), smoking history, and alcohol consumption history. Additionally, we gathered tumor characteristics such as tumor location, pathological tumor stage (ypT), pathological lymph node status (ypN), tumor differentiation, nerve invasion, and vessel invasion.

All hematoxylin–eosin‐stained sections, including margins and lymph nodes, were examined by pathologists who were unaware of the treatment or outcomes. Tumor staging was based on the depth of invasion and lymph node metastasis, using the Eighth edition of the AJCC staging system [16]. Tumor response was evaluated using TRG, which was determined by the estimated percentage of viable residual tumor cells (VRTCs): Grade 0, pathological complete response; Grade 1, nearly complete response with < 10% VRTCs; Grade 2, partial response with 10%–50% VRTCs; and Grade 3, > 50% VRTCs. Patients were categorized as good responders (TRG 0–1) and poor responders (TRG 2–3) [11].

### 2.3 Statistical Analysis

Statistical analyses were performed using R software (version 4.1.0, R Foundation for Statistical Computing, Vienna, Austria). Continuous variables were presented as mean ± standard deviation or median (interquartile range), and categorical variables as frequencies and percentages. Descriptive statistics were calculated using *t*‐tests, Wilcoxon rank‐sum tests, chi‐square tests, or Fisher's exact tests, as appropriate. Survival analysis was conducted using the Kaplan–Meier method to estimate survival curves, with differences between groups compared using the log‐rank test. Cox proportional hazards regression models were used to calculate hazard ratios and 95% confidence intervals to assess the impact of various variables on survival time. Forest plots and survival analysis were generated using the “forestplot” and “survival” packages in R, respectively. All statistical tests were two‐sided, with a significance level set at *p* < 0.05.

## 3 Results

### 3.1 Patient Characteristics

After applying the inclusion and exclusion criteria, the study included 416 patients who underwent NAT followed by radical esophagectomy for esophageal cancer. Table 1 shows the baseline characteristics of the patients. Among them, 269 patients (64.7%) received neoadjuvant chemotherapy, 77 patients (18.5%) received neoadjuvant chemoradiotherapy, and 70 patients (16.8%) received combined neoadjuvant chemotherapy and immunotherapy. A total of 253 patients (60.8%) received postoperative adjuvant therapy, with the majority (87.0%) receiving adjuvant chemotherapy, while a smaller proportion (13.0%) received a combination of chemotherapy and immunotherapy. The cohort was predominantly male (80.0%), with a median age of 60 years (range 38 to 78 years). The median follow‐up time was 52 months (interquartile range 37–72 months).

**TABLE 1:** Baseline characteristics of the study patients.

Variables	TRG 0–1 (*n* = 147)	TRG 0–1		*p*	TRG 2–3 (*n* = 269)	TRG 2–3		*p*
		None (*n* = 68)	Adjuvant therapy (*n* = 79)			None (*n* = 95)	Adjuvant therapy (*n* = 174)	
Age, Mean ± SD	60.62 ± 7.09	61.16 ± 7.67	60.15 ± 6.56	0.391	59.65 ± 7.67	60.29 ± 7.88	59.30 ± 7.56	0.310
BMI, Mean ± SD	22.19 ± 2.77	21.95 ± 2.54	22.40 ± 2.95	0.320	21.76 ± 3.07	21.64 ± 3.10	21.83 ± 3.06	0.633
Gender, *n* (%)				0.960				0.681
Male	117 (79.59)	54 (79.41)	63 (79.75)		216 (80.30)	75 (78.95)	141 (81.03)	
Female	30 (20.41)	14 (20.59)	16 (20.25)		53 (19.70)	20 (21.05)	33 (18.97)	
Location, *n* (%)				0.966				0.541
Upper	23 (15.65)	11 (16.18)	12 (15.19)		43 (15.99)	12 (12.63)	31 (17.82)	
Middle	86 (58.50)	39 (57.35)	47 (59.49)		155 (57.62)	57 (60.00)	98 (56.32)	
Lower	38 (25.85)	18 (26.47)	20 (25.32)		71 (26.39)	26 (27.37)	45 (25.86)	
cT, *n* (%)				0.622				0.271
T2	44 (29.93)	23 (33.82)	21 (26.58)		67 (24.91)	28 (29.47)	39 (22.41)	
T3	63 (42.86)	28 (41.18)	35 (44.30)		139 (51.67)	43 (45.26)	96 (55.17)	
T4	40 (27.21)	17 (25.00)	23 (29.11)		63 (23.42)	24 (25.26)	39 (22.41)	
cN, *n* (%)				0.561				0.707
N0	33 (22.45)	16 (23.53)	17 (21.52)		39 (14.50)	16 (16.84)	23 (13.22)	
N1	36 (24.49)	13 (19.12)	23 (29.11)		78 (29.00)	26 (27.37)	52 (29.89)	
N2	51 (34.69)	26 (38.24)	25 (31.65)		115 (42.75)	38 (40.00)	77 (44.25)	
N3	27 (18.37)	13 (19.12)	14 (17.72)		37 (13.75)	15 (15.79)	22 (12.64)	
ypT, *n* (%)				0.039				0.562
T0	48 (32.65)	26 (38.24)	22 (27.85)		3 (1.12)	2 (2.11)	1 (0.57)	
T1	48 (32.65)	27 (39.71)	21 (26.58)		20 (7.43)	9 (9.47)	11 (6.32)	
T2	28 (19.05)	9 (13.24)	19 (24.05)		50 (18.59)	18 (18.95)	32 (18.39)	
T3	20 (13.61)	6 (8.82)	14 (17.72)		184 (68.40)	61 (64.21)	123 (70.69)	
T4	3 (2.04)	0 (0.00)	3 (3.80)		12 (4.46)	5 (5.26)	7 (4.02)	
ypN, *n* (%)				0.730				0.041
N0	102 (69.39)	50 (73.53)	52 (65.82)		104 (38.66)	46 (48.42)	58 (33.33)	
N1	29 (19.73)	11 (16.18)	18 (22.78)		90 (33.46)	27 (28.42)	63 (36.21)	
N2	13 (8.84)	6 (8.82)	7 (8.86)		57 (21.19)	14 (14.74)	43 (24.71)	
N3	3 (2.04)	1 (1.47)	2 (2.53)		18 (6.69)	8 (8.42)	10 (5.75)	
Differentiation, *n* (%)				0.292				0.129
G1	65 (44.22)	32 (47.06)	33 (41.77)		87 (32.34)	28 (29.47)	59 (33.91)	
G2	59 (40.14)	23 (33.82)	36 (45.57)		138 (51.30)	56 (58.95)	82 (47.13)	
G3 or x	23 (15.65)	13 (19.12)	10 (12.66)		44 (16.36)	11 (11.58)	33 (18.97)	
Vessel invasion, *n* (%)				0.722				0.770
No	143 (97.28)	67 (98.53)	76 (96.20)		227 (84.39)	81 (85.26)	146 (83.91)	
Yes	4 (2.72)	1 (1.47)	3 (3.80)		42 (15.61)	14 (14.74)	28 (16.09)	
Nerve invasion, *n* (%)				1.000				0.035
No	142 (96.60)	66 (97.06)	76 (96.20)		210 (78.07)	81 (85.26)	129 (74.14)	
Yes	5 (3.40)	2 (2.94)	3 (3.80)		59 (21.93)	14 (14.74)	45 (25.86)	
Smoking, *n* (%)				0.880				0.475
No	55 (37.41)	25 (36.76)	30 (37.97)		104 (38.66)	34 (35.79)	70 (40.23)	
Yes	92 (62.59)	43 (63.24)	49 (62.03)		165 (61.34)	61 (64.21)	104 (59.77)	
Drinking, *n* (%)				0.978				0.781
No	69 (46.94)	32 (47.06)	37 (46.84)		130 (48.33)	47 (49.47)	83 (47.70)	
Yes	78 (53.06)	36 (52.94)	42 (53.16)		139 (51.67)	48 (50.53)	91 (52.30)	
Neoadjuvant therapy, *n* (%)				0.091				0.017
Immunochemotherapy	30 (20.41)	15 (22.06)	15 (18.99)		40 (14.87)	18 (18.95)	22 (12.64)	
Chemoradiotherapy	45 (30.61)	26 (38.24)	19 (24.05)		32 (11.90)	17 (17.89)	15 (8.62)	
Chemotherapy	72 (48.98)	27 (39.71)	45 (56.96)		197 (73.23)	60 (63.16)	137 (78.74)	
Robotassisted, *n* (%)				0.863				0.599
No	118 (80.27)	55 (80.88)	63 (79.75)		228 (84.76)	82 (86.32)	146 (83.91)	
Yes	29 (19.73)	13 (19.12)	16 (20.25)		41 (15.24)	13 (13.68)	28 (16.09)	
Lymphadenectomy, *n* (%)				0.441				0.566
Two‐field	117 (79.59)	56 (82.35)	61 (77.22)		207 (76.95)	75 (78.95)	132 (75.86)	
Three‐field	30 (20.41)	12 (17.65)	18 (22.78)		62 (23.05)	20 (21.05)	42 (24.14)	
Anastomoticleakage, *n* (%)				0.177				0.362
No	129 (87.76)	57 (83.82)	72 (91.14)		236 (87.73)	81 (85.26)	155 (89.08)	
Yes	18 (12.24)	11 (16.18)	7 (8.86)		33 (12.27)	14 (14.74)	19 (10.92)	

Among the 147 patients with TRG 0–1, 79 (53.7%) received adjuvant therapy. Among those who received adjuvant therapy, 21.5% were classified as ypT3‐4 stage, in contrast to only 8.82% among those who did not. For the 269 patients with TRG 2–3, 174 (64.7%) received adjuvant therapy. Among the patients who received adjuvant therapy, 66.7% had positive lymph nodes, and 26.01% had nerve invasion.

### 3.2 Impact of Adjuvant Therapy on Overall Survival

**FIGURE 2:**
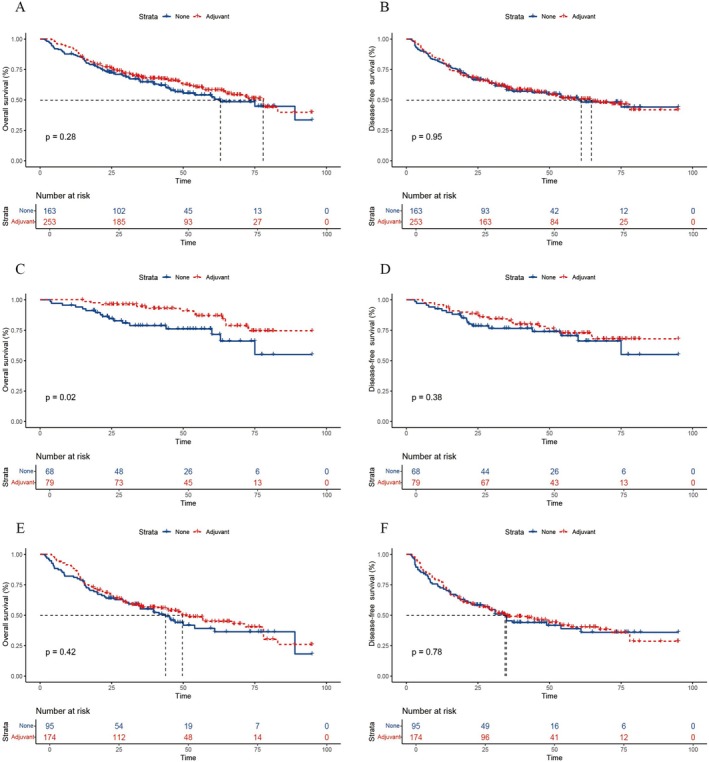
Comparison of overall survival (OS) and disease‐free survival (DFS) between adjuvant and non‐adjuvant treatment groups. (A) OS in the overall patient population. (B) DFS in the overall patient population. (C) OS in patients with TRG grades 0–1. (D) DFS in patients with TRG grades 0–1. (E) OS in patients with TRG grades 2–3. (F) DFS in patients with TRG grades 2–3.

The median survival for patients receiving adjuvant therapy was 78 months, versus 70 months for those without adjuvant therapy, reflecting an 8‐month increase. However, this difference was not statistically significant (*p* = 0.28). The 3‐year and 5‐year survival rates were 68.2% and 58%, respectively, for the adjuvant therapy group, compared to 64.8% and 54% for the non‐adjuvant therapy group (Figure 2A). Notably, there was no difference in disease‐free survival (DFS) between the adjuvant and non‐adjuvant therapy groups (Figure 2B).

Among patients with TRG grades 0–1, the 3‐year and 5‐year survival rates were 94.6% and 86.8% for the adjuvant therapy group, compared to 78.8% and 71.6% for the non‐adjuvant therapy group (*p* = 0.02) (Figure 2C). Notably, DFS did not differ significantly between these groups (Figure 2D). For patients with TRG grades 2–3, the median survival was 49.6 months for those receiving adjuvant therapy, compared to 43.8 months for those without, indicating an increase of 5.8 months. However, this difference was not statistically significant (*p* = 0.42) (Figure 2E). Similarly, DFS was comparable between the adjuvant and non‐adjuvant groups in this subset (Figure 2F).

### 3.3 Univariate and Multivariate Analyses for Overall Survival in Subgroups

A Cox proportional hazards model was used to identify variables independently associated with the risk of overall mortality (Table 2). In the TRG 0–1 subgroup, after adjusting for other patient and tumor factors, receiving adjuvant therapy was associated with a reduced risk of mortality (HR, 0.32; 95% CI, 0.14–0.73; *p* = 0.006). Other variables independently associated with reduced mortality included normal weight (HR, 0.30; 95% CI, 0.11–0.82; *p* = 0.019) and overweight (HR, 0.24; 95% CI, 0.06–0.90; *p* = 0.035). In the TRG 2–3 subgroup, receiving adjuvant therapy was not associated with a reduction in mortality. The only variable independently associated with reduced mortality in this subgroup was being female (HR, 0.56; 95% CI, 0.34–0.91; *p* = 0.020).

**TABLE 2:** Cox proportional hazards model for variables independently associated with overall survival after esophagectomy in different TRG groups.

Variables	TRG 0–1				TRG 2–3			
	Univariate analysis		Multivariate analysis		Univariate analysis		Multivariate analysis	
	HR (95% CI)	*p*	HR (95% CI)	*p*	HR (95% CI)	*p*	HR (95% CI)	*p*
ypT								
T0‐2	1.00 (Reference)				1.00 (Reference)		1.00 (Reference)	
T3‐4	1.20 (0.49–2.97)	0.686			1.71 (1.14–2.58)	**0.010**	1.59 (1.05–2.41)	**0.030**
ypN								
N0	1.00 (Reference)		1.00 (Reference)		1.00 (Reference)		1.00 (Reference)	
N1	1.24 (0.45–3.40)	0.674	1.97 (0.68–5.70)	0.210	1.73 (1.15–2.61)	**0.009**	1.53 (1.01–2.33)	**0.047**
N2	3.67 (1.43–9.44)	**0.007**	4.88 (1.72–13.86)	**0.003**	1.84 (1.17–2.89)	**0.009**	1.65 (1.03–2.65)	**0.037**
N3	3.91 (0.90–17.10)	0.070	7.27 (1.49–35.55)	**0.014**	2.86 (1.58–5.20)	**< 0.001**	2.24 (1.14–4.37)	**0.019**
Differentiation								
G1	1.00 (Reference)		1.00 (Reference)		1.00 (Reference)			
G2	1.85 (0.73–4.71)	0.195	1.81 (0.68–4.82)	0.238	1.08 (0.74–1.57)	0.700		
G3 or x	4.54 (1.73–11.92)	**0.002**	3.68 (1.33–10.17)	**0.012**	1.32 (0.81–2.16)	0.263		
Adjuvant								
No	1.00 (Reference)		1.00 (Reference)		1.00 (Reference)			
Yes	0.42 (0.20–0.89)	**0.023**	0.32 (0.14–0.73)	**0.006**	0.87 (0.62–1.22)	0.423		
BMI								
< 19	1.00 (Reference)		1.00 (Reference)		1.00 (Reference)			
19–25	0.41 (0.16–1.04)	0.060	0.30 (0.11–0.82)	**0.019**	1.02 (0.66–1.57)	0.933		
≥ 25	0.31 (0.09–1.10)	0.070	0.24 (0.06–0.90)	**0.035**	1.28 (0.71–2.31)	0.414		
Vessel invasion								
No	1.00 (Reference)				1.00 (Reference)		1.00 (Reference)	
Yes	0.00 (0.00–Inf)	0.997			1.53 (1.01–2.34)	**0.047**	1.08 (0.67–1.75)	0.757
Nerve invasion								
No	1.00 (Reference)				1.00 (Reference)			
Yes	2.51 (0.59–10.59)	0.212			1.29 (0.87–1.91)	0.200		
Location								
Upper	1.00 (Reference)				1.00 (Reference)		1.00 (Reference)	
Middle	0.69 (0.29–1.66)	0.412			1.43 (0.88–2.32)	0.146	1.31 (0.80–2.15)	0.291
Lower	0.37 (0.11–1.27)	0.114			1.59 (0.93–2.71)	0.088	1.39 (0.81–2.38)	0.237
Age								
< 60	1.00 (Reference)				1.00 (Reference)			
≥ 60	1.59 (0.75–3.39)	0.230			1.00 (0.70–1.43)	0.999		
Gender								
Male	1.00 (Reference)				1.00 (Reference)		1.00 (Reference)	
Female	0.88 (0.33–2.33)	0.802			0.53 (0.32–0.85)	**0.009**	0.56 (0.34–0.91)	**0.020**

*Note:* Bold values indicate statistical significance (*p* < 0.05).

Abbreviations: CI, Confidence Interval; HR, Hazards Ratio.

For patients with positive postoperative lymph nodes, advanced T stage was identified as an independent risk factor, whereas postoperative adjuvant therapy served as an independent protective factor. In the YpT3‐4 subgroup, both positive postoperative lymph nodes and TRG grades 2–3 were independent risk factors, while postoperative adjuvant therapy remained a protective factor. Conversely, in subgroups with negative postoperative lymph nodes and those with YpT2‐3 stage, the Cox proportional hazards model showed that adjuvant therapy was not associated with reduced mortality (Tables S1 and S2).

### 3.4 Heterogeneity of Treatment Effects Based on Baseline Characteristics Stratified by TRG Groups

**FIGURE 3:**
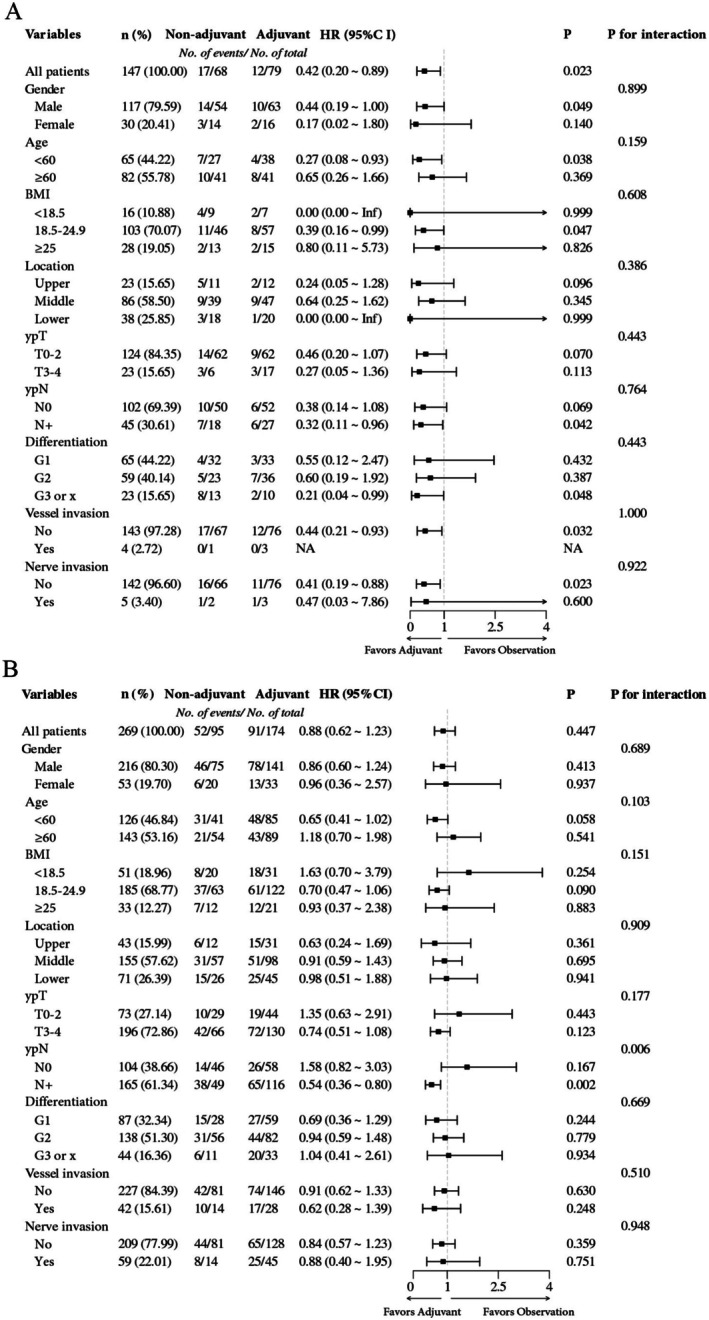
Heterogeneity of treatment effects based on baseline characteristics stratified by TRG groups. (A) Heterogeneity of treatment effects in TRG 0–1 patients. (B) Heterogeneity of treatment effects in TRG 2–3 patients.

Figure 3 shows that within the TRG 0–1 subgroup, there was no clear evidence of heterogeneity of treatment effect according to sex, age group, BMI category, tumor location, postoperative *T* stage, postoperative *N* stage or tumor differentiation. In the TRG 2–3 subgroup, survival benefits from adjuvant therapy were observed only in the subgroup with positive pathological lymph nodes, with no evidence of benefit in the other groups.

## 4 Discussion

Neoadjuvant therapy is widely acknowledged as effective in treating esophageal cancer [17, 18, 19], but the role of postoperative adjuvant therapy remains debated due to insufficient data to determine which patients might benefit or be harmed [20, 21, 22]. To the best of our knowledge, this is the first study to demonstrate the significant value of TRG in predicting the efficacy of adjuvant therapy in patients with ESCC following NAT. We selected patients from three medical centers that uniformly employed the same regimen—platinum plus paclitaxel. This approach not only increased our sample size but also reduced potential bias arising from regimen heterogeneity.

In this multicenter study, although adjuvant therapy extended median survival by 8 months, the difference did not reach statistical significance. However, subgroup analyses revealed significant survival benefits in patients with TRG 0–1 and those with ypN+ and ypT3‐4 disease, in contrast to the absence of benefit in patients with TRG 2–3. This differential response pattern underscores the potential predictive value of TRG, particularly given that TRG 2–3 patients constituted 64.7% (269/416) of the cohort, likely attenuating the overall treatment effect. Previous retrospective studies have provided mixed conclusions. One study involving 209 patients from nine institutions found that adjuvant therapy independently improved survival, reducing mortality by 24% (HR = 0.76, *p* = 0.005) [23]. Brescia et al., in a study of 101 patients, noted that those receiving adjuvant therapy had better median survival compared to those who did not (24.0 vs. 18.0 months, *p* = 0.033) [5]. In contrast, Stiles et al. found that adjuvant therapy was not a significant prognostic factor for overall survival (*p* = 0.9), with clinical and pathological staging and the number of positive lymph nodes emerging as more important predictors [24]. These findings highlighted the importance of patient selection in assessing the benefits of adjuvant therapy.

There are notable individual differences in how patients respond to NAT, which poses challenges for deciding on postoperative treatment. TRG has emerged as a key measure for evaluating NAT responses. Several studies have investigated the role of TRG in predicting the efficacy of adjuvant therapy for esophageal adenocarcinoma, but have reached controversial conclusions [14, 25]. A European study involving 134 esophageal adenocarcinoma patients found that adjuvant chemotherapy improved survival in those with minimal or no tumor regression following NAT [25]. Conversely, a UK study suggested that only patients who responded histopathologically to NAT benefited from adjuvant chemotherapy, while nonresponders did not benefit and might have even experienced higher mortality [14]. Yet, no studies have specifically explored using TRG to guide treatment in ESCC.

In this study, patients with a good TRG response benefited significantly from adjuvant therapy, whereas patients with a poor response did not. This finding contrasts with common clinical practices, where adjuvant therapy was used in patients with potential residual disease after NAT in the hope of eradicating microscopic residuals and improving survival. The proportion of patients receiving postoperative adjuvant therapy was significantly higher in patients with positive lymph nodes or higher *T*‐stages [14, 25], and similar results were found in our study. By the same token, postoperative adjuvant therapy was more frequently used in populations with a poor TRG response, as these patients have a higher likelihood of potential residual disease. However, the results of our study suggested that postoperative adjuvant therapy did not improve the survival of such patients. In addition, for patients with complete responses, observation is typically recommended according to NCCN guidelines [4]. However, a significant survival benefit can be observed with adjuvant therapy in patients with a good TRG response (including complete pathological remission and < 10% residual tumor). We believe that TRG accurately reflects an individual's response to NAT and therefore has significant value in predicting the efficacy of adjuvant therapy. This finding may provide a new perspective for further research on adjuvant therapy for ESCC.

Our study found that patients with positive lymph nodes experienced significantly prolonged survival following adjuvant therapy. This suggests that lymph node status could be a critical factor in determining optimal adjuvant therapy strategies. Supporting literature also indicates that patients with positive lymph nodes generally have poorer prognoses and may benefit from additional adjuvant therapy [5]. Samson et al. demonstrated that patients who received adjuvant chemotherapy after induction therapy and esophagectomy showed survival benefits across all lymph node‐positive stages [26]. Bur et al. indicated that adjuvant chemotherapy following neoadjuvant chemoradiotherapy and esophagectomy improved survival rates in patients with residual nodal disease [6]. Furthermore, the impact of tumor T‐stage on the efficacy of adjuvant therapy should not be underestimated. Our study revealed that patients with T3–T4 stages had significantly prolonged survival following adjuvant therapy. This finding aligns with previous research suggesting that patients with advanced‐stage tumors may derive greater benefit from aggressive treatment approaches [7, 8, 9].

Despite its insights, this study has several limitations. It included 416 patients, but the small sample sizes in certain subgroups may undermine the statistical robustness and the applicability of the findings. Moreover, the study did not thoroughly analyze the reasons why some postoperative patients did not receive adjuvant therapy, such as age, physical condition, or postoperative complications. Additionally, our conclusions rely on retrospective data, highlighting the need for prospective studies to validate the benefits of adjuvant therapy across different patient subgroups. Lastly, this study did not consider molecular biological markers that could potentially predict the efficacy of adjuvant therapy.

## 5 Conclusion

In esophageal cancer, adjuvant therapy generally has a limited impact on overall survival but can significantly benefit specific subgroups. TRG is a promising predictor of the prognostic value of adjuvant therapy in ESCC patients. Patients with a good TRG response, positive lymph nodes, and ypT3‐4 stage benefit from adjuvant therapy. More research is necessary to confirm these findings and support clinical decision‐making.

## Author Contributions

**Yizhou Huang:** conceptualization, formal analysis, visualization, writing – original draft. **Maohui Chen:** conceptualization, formal analysis, visualization, writing – original draft. **Yuanpu Wei:** data curation, investigation. **Bingqiang Cai:** data curation. **Yongcong Zhang:** data curation. **Chuanquan Lin:** investigation. **Shuliang Zhang:** resources. **Taidui Zeng:** resources. **Chun Chen:** conceptualization, supervision, writing – review and editing. **Bin Zheng:** conceptualization, supervision, writing – review and editing.

## Ethics Statement

This retrospective study was conducted in accordance with the Declaration of Helsinki (as revised in 2013) and was approved by the Ethics Committee of Fujian Medical University Union Hospital (2024KY133). The requirement of informed consent from the study participants was waived due to the retrospective nature of this study.

## Conflicts of Interest

The authors declare no conflicts of interest.

## Supporting information


**Table S1:** Cox proportional hazards model for variables independently associated with overall survival after esophagectomy in different YpN groups.

**Table S2:** Cox proportional hazards model for variables independently associated with overall survival after esophagectomy in different YpT groups.

## Data Availability

The data presented in this study are available on request from the corresponding author.
