# Supplementary material for: Tumor Regression Grade as a Predictor of Adjuvant Therapy Benefits in Esophageal Squamous Cell Carcinoma Patients After Neoadjuvant Therapy
Source: Cancer Med. 2025 Sep 13;14(18):e71166. doi: 10.1002/cam4.71166 (PMC12432406; doi:10.1002/cam4.71166)
Supplement: Supplementary file 1 — Table S1: Cox proportional hazards model for variables independently associated with overall survival after esophagectomy in different YpN groups. [file CAM4-14-e71166-s002.docx]

Supplementary Table 1 Cox proportional hazards model for variables independently associated with overall survival after esophagectomy in different YpN groups.

| Variables | ypN0 | | | | |  | ypN+ | | | | |
| --- | --- | --- | --- | --- | --- | --- | --- | --- | --- | --- | --- |
|  |  | Univariate analysis |  | Multivariate analysis |  |  |  | Univariate analysis |  | Multivariate analysis |  |
|  |  | HR (95%CI) | *P* | HR (95%CI) | *P* |  |  | HR (95%CI) | *P* | HR (95%CI) | *P* |
| ypT |  |  |  |  |  |  |  |  |  |  |  |
| T0-2 |  | 1.00 (Reference) |  | 1.00 (Reference) |  |  |  | 1.00 (Reference) |  | 1.00 (Reference) |  |
| T3-4 |  | 1.64 (0.97 ~ 2.77) | 0.063 | 0.84 (0.46 ~ 1.54) | 0.581 |  |  | 2.57 (1.64 ~ 4.03) | **<.001** | 1.89 (1.13 ~ 3.18) | **0.016** |
| Differentiation |  |  |  |  |  |  |  |  |  |  |  |
| G1 |  | 1.00 (Reference) |  | 1.00 (Reference) |  |  |  | 1.00 (Reference) |  |  |  |
| G2 |  | 1.53 (0.83 ~ 2.82) | 0.178 | 1.38 (0.74 ~ 2.57) | 0.311 |  |  | 1.18 (0.77 ~ 1.81) | 0.440 |  |  |
| G3 or x |  | 2.26 (1.07 ~ 4.79) | **0.033** | 2.45 (1.14 ~ 5.26) | **0.022** |  |  | 1.45 (0.86 ~ 2.46) | 0.166 |  |  |
| Adjuvant |  |  |  |  |  |  |  |  |  |  |  |
| No |  | 1.00 (Reference) |  |  |  |  |  | 1.00 (Reference) |  | 1.00 (Reference) |  |
| Yes |  | 1.07 (0.63 ~ 1.82) | 0.808 |  |  |  |  | 0.54 (0.37 ~ 0.79) | **0.002** | 0.53 (0.36 ~ 0.78) | **0.001** |
| Gender |  |  |  |  |  |  |  |  |  |  |  |
| Male |  | 1.00 (Reference) |  |  |  |  |  | 1.00 (Reference) |  | 1.00 (Reference) |  |
| Female |  | 0.69 (0.34 ~ 1.41) | 0.307 |  |  |  |  | 0.55 (0.32 ~ 0.95) | **0.033** | 0.63 (0.36 ~ 1.09) | 0.098 |
| Age |  |  |  |  |  |  |  |  |  |  |  |
| <60 |  | 1.00 (Reference) |  |  |  |  |  | 1.00 (Reference) |  |  |  |
| ≥60 |  | 1.07 (0.63 ~ 1.82) | 0.802 |  |  |  |  | 0.79 (0.54 ~ 1.14) | 0.211 |  |  |
| BMI |  |  |  |  |  |  |  |  |  |  |  |
| ＜19 |  | 1.00 (Reference) |  |  |  |  |  | 1.00 (Reference) |  |  |  |
| 19-25 |  | 0.94 (0.46 ~ 1.95) | 0.876 |  |  |  |  | 0.72 (0.45 ~ 1.14) | 0.163 |  |  |
| ≥25 |  | 0.88 (0.34 ~ 2.29) | 0.798 |  |  |  |  | 0.74 (0.39 ~ 1.42) | 0.367 |  |  |
| Location |  |  |  |  |  |  |  |  |  |  |  |
| Upper |  | 1.00 (Reference) |  | 1.00 (Reference) |  |  |  | 1.00 (Reference) |  | 1.00 (Reference) |  |
| Middle |  | 0.69 (0.37 ~ 1.28) | 0.241 | 0.64 (0.34 ~ 1.21) | 0.170 |  |  | 1.79 (0.97 ~ 3.29) | 0.061 | 1.58 (0.85 ~ 2.95) | 0.147 |
| Lower |  | 0.50 (0.22 ~ 1.12) | 0.091 | 0.45 (0.20 ~ 1.00) | 0.051 |  |  | 2.14 (1.12 ~ 4.09) | **0.022** | 1.75 (0.90 ~ 3.40) | 0.098 |
| TRG |  |  |  |  |  |  |  |  |  |  |  |
| 0-1 |  | 1.00 (Reference) |  | 1.00 (Reference) |  |  |  | 1.00 (Reference) |  | 1.00 (Reference) |  |
| 2-3 |  | 3.05 (1.70 ~ 5.45) | **<.001** | 3.32 (1.71 ~ 6.43) | **<.001** |  |  | 2.77 (1.56 ~ 4.94) | **<.001** | 1.92 (0.99 ~ 3.70) | 0.053 |
| Vesselinvasion |  |  |  |  |  |  |  |  |  |  |  |
| No |  | 1.00 (Reference) |  | 1.00 (Reference) |  |  |  | 1.00 (Reference) |  |  |  |
| Yes |  | 2.79 (1.01 ~ 7.73) | **0.049** | 3.39 (1.17 ~ 9.83) | **0.024** |  |  | 1.25 (0.79 ~ 1.98) | 0.338 |  |  |
| Nerveinvasion |  |  |  |  |  |  |  |  |  |  |  |
| No |  | 1.00 (Reference) |  |  |  |  |  | 1.00 (Reference) |  | 1.00 (Reference) |  |
| Yes |  | 0.89 (0.28 ~ 2.85) | 0.846 |  |  |  |  | 1.55 (1.03 ~ 2.34) | **0.035** | 1.27 (0.82 ~ 1.97) | 0.289 |
| HR: Hazards Ratio, CI: Confidence Interval | | | | | | | | | | | |
