# Supplementary material for: Tumor Regression Grade as a Predictor of Adjuvant Therapy Benefits in Esophageal Squamous Cell Carcinoma Patients After Neoadjuvant Therapy
Source: Cancer Med. 2025 Sep 13;14(18):e71166. doi: 10.1002/cam4.71166 (PMC12432406; doi:10.1002/cam4.71166)
Supplement: Supplementary file 2 — Table S2: Cox proportional hazards model for variables independently associated with overall survival after esophagectomy in different YpT groups. [file CAM4-14-e71166-s001.docx]

Supplementary Table 2 Cox proportional hazards model for variables independently associated with overall survival after esophagectomy in different YpT groups.

| Variables | YpT0-2 | | | | |  | YpT3-4 | | | | |
| --- | --- | --- | --- | --- | --- | --- | --- | --- | --- | --- | --- |
|  |  | Univariate analysis |  | Multivariate analysis |  |  |  | Univariate analysis |  | Multivariate analysis |  |
|  |  | HR (95%CI) | *P* | HR (95%CI) | *P* |  |  | HR (95%CI) | *P* | HR (95%CI) | *P* |
| ypN |  |  |  |  |  |  |  |  |  |  |  |
| N0 |  | 1.00 (Reference) |  | 1.00 (Reference) |  |  |  | 1.00 (Reference) |  | 1.00 (Reference) |  |
| N1 |  | 0.90 (0.41 ~ 1.99) | 0.801 | 0.85 (0.38 ~ 1.89) | 0.696 |  |  | 2.36 (1.49 ~ 3.76) | **<.001** | 2.12 (1.32 ~ 3.41) | **0.002** |
| N2 |  | 2.91 (1.47 ~ 5.74) | **0.002** | 1.77 (0.82 ~ 3.84) | 0.146 |  |  | 2.26 (1.34 ~ 3.80) | **0.002** | 2.18 (1.28 ~ 3.71) | **0.004** |
| N3 |  | 3.41 (1.19 ~ 9.74) | **0.022** | 3.28 (1.14 ~ 9.45) | **0.028** |  |  | 3.48 (1.79 ~ 6.75) | **<.001** | 2.70 (1.38 ~ 5.31) | **0.004** |
| Differentiation |  |  |  |  |  |  |  |  |  |  |  |
| G1 |  | 1.00 (Reference) |  | 1.00 (Reference) |  |  |  | 1.00 (Reference) |  |  |  |
| G2 |  | 2.06 (1.04 ~ 4.08) | **0.039** | 1.95 (0.97 ~ 3.92) | 0.062 |  |  | 1.00 (0.67 ~ 1.51) | 0.994 |  |  |
| G3 or x |  | 3.80 (1.76 ~ 8.22) | **<.001** | 3.40 (1.53 ~ 7.57) | **0.003** |  |  | 1.17 (0.69 ~ 1.98) | 0.571 |  |  |
| Adjuvant |  |  |  |  |  |  |  |  |  |  |  |
| No |  | 1.00 (Reference) |  |  |  |  |  | 1.00 (Reference) |  | 1.00 (Reference) |  |
| Yes |  | 0.86 (0.50 ~ 1.49) | 0.588 |  |  |  |  | 0.68 (0.47 ~ 0.98) | **0.038** | 0.63 (0.43 ~ 0.92) | **0.017** |
| Gender |  |  |  |  |  |  |  |  |  |  |  |
| Male |  | 1.00 (Reference) |  |  |  |  |  | 1.00 (Reference) |  | 1.00 (Reference) |  |
| Female |  | 0.49 (0.20 ~ 1.25) | 0.136 |  |  |  |  | 0.55 (0.34 ~ 0.90) | **0.017** | 0.63 (0.38 ~ 1.03) | 0.067 |
| Age |  |  |  |  |  |  |  |  |  |  |  |
| <60 |  | 1.00 (Reference) |  |  |  |  |  | 1.00 (Reference) |  |  |  |
| ≥60 |  | 0.93 (0.54 ~ 1.61) | 0.801 |  |  |  |  | 0.77 (0.54 ~ 1.11) | 0.165 |  |  |
| BMI |  |  |  |  |  |  |  |  |  |  |  |
| ＜19 |  | 1.00 (Reference) |  |  |  |  |  | 1.00 (Reference) |  |  |  |
| 19-25 |  | 0.91 (0.42 ~ 1.96) | 0.809 |  |  |  |  | 0.79 (0.50 ~ 1.26) | 0.324 |  |  |
| ≥25 |  | 0.68 (0.25 ~ 1.87) | 0.454 |  |  |  |  | 0.95 (0.50 ~ 1.79) | 0.870 |  |  |
| Location |  |  |  |  |  |  |  |  |  |  |  |
| Upper |  | 1.00 (Reference) |  |  |  |  |  | 1.00 (Reference) |  |  |  |
| Middle |  | 0.81 (0.40 ~ 1.64) | 0.562 |  |  |  |  | 1.41 (0.83 ~ 2.39) | 0.209 |  |  |
| Lower |  | 1.01 (0.46 ~ 2.21) | 0.977 |  |  |  |  | 1.42 (0.78 ~ 2.59) | 0.252 |  |  |
| TRG |  |  |  |  |  |  |  |  |  |  |  |
| 0-1 |  | 1.00 (Reference) |  | 1.00 (Reference) |  |  |  | 1.00 (Reference) |  | 1.00 (Reference) |  |
| 2-3 |  | 2.62 (1.51 ~ 4.54) | **<.001** | 2.34 (1.28 ~ 4.26) | **0.005** |  |  | 3.29 (1.44 ~ 7.49) | **0.005** | 2.37 (1.02 ~ 5.48) | **0.044** |
| Vesselinvasion |  |  |  |  |  |  |  |  |  |  |  |
| No |  | 1.00 (Reference) |  |  |  |  |  | 1.00 (Reference) |  |  |  |
| Yes |  | 1.85 (0.57 ~ 5.96) | 0.306 |  |  |  |  | 1.41 (0.90 ~ 2.21) | 0.136 |  |  |
| Nerveinvasion |  |  |  |  |  |  |  |  |  |  |  |
| No |  | 1.00 (Reference) |  |  |  |  |  | 1.00 (Reference) |  |  |  |
| Yes |  | 3.10 (0.74 ~ 12.90) | 0.121 |  |  |  |  | 1.18 (0.79 ~ 1.77) | 0.410 |  |  |
| HR: Hazards Ratio, CI: Confidence Interval | | | | | | | | | | | |
